# The Corrosion Behavior of Pure Iron under Solid Na_2_SO_4_ Deposit in Wet Oxygen Flow at 500 °C

**DOI:** 10.3390/ma7096144

**Published:** 2014-08-27

**Authors:** Yanbing Tang, Li Liu, Lei Fan, Ying Li, Fuhui Wang

**Affiliations:** 1 Institute of Metal Research, Chinese Academy of Sciences, Wencui Road 62, Shenyang 110016, Liaoning, China; E-Mails: tyanbing@gzpcc.com (Y.T.); lfan10s@imr.ac.cn (L.F.); fhwang@imr.ac.cn (F.W.); 2 Key Laboratory of Durability Technology for Harbor and Marine Structure Ministry of Communications, CCCC Fourth Harbor Engineering Institute Co., Ltd., Qianjin Road 157, Guangzhou 510230, Guangdong, China

**Keywords:** electrochemical reaction, Na_2_SO_4_ + O_2_ + H_2_O, corrosion behavior

## Abstract

The corrosion behavior of pure Fe under a Na_2_SO_4_ deposit in an atmosphere of O_2_ + H_2_O was investigated at 500 °C by thermo gravimetric, and electrochemical measurements, *viz*. potentiodynamic polarization, electrochemical impedance spectroscopy (EIS), and surface characterization methods *viz*. X-ray diffraction (XRD), and scanning electron microscope (SEM)/energy dispersive spectroscopy(EDS). The results showed that a synergistic effect occurred between Na_2_SO_4_ and O_2_ + H_2_O, which significantly accelerated the corrosion rate of the pure Fe. Briefly, NaFeO_2_ was formed in addition to the customary Fe oxides; at the same time, H_2_SO_4_ gas was produced by introduction of water vapor. Subsequently, an electrochemical corrosion reaction occurred due to the existence of Na_2_SO_4_, NaFeO_2_, and H_2_O. When this coupled to the chemical corrosion reaction, the progress of the chemical corrosion reaction was promoted and eventually resulted in the acceleration of the corrosion of the pure Fe.

## 1. Introduction

In the last decade, it has been found that the corrosion of some pure metals and their alloys such as 1Cr11NiW2MoV, Fe-5Cr, Fe-15Cr, and Fe-25Cr (mass percent) was accelerated under solid NaCl in an atmosphere of H_2_O + O_2_ at intermediate temperatures (300–600 °C) [1,2,3,4,5,6,7,8]. This phenomenon has been notably reflected in the corrosion of turbine blades in aeroplanes serving in the vicinity of the sea. Actually, a synergistic effect, which is prone to accelerate the corrosion of metals/alloys, was observed when solid salt such as NaCl and water vapor (H_2_O) were used together at 300–600 °C [1,2,3,4,5,6,7,8]. Shu *et al.* [1,2,3,4] found that the involvement of H_2_O significantly accelerated the corrosion rate of many pure metals and alloys. This is especially the case for pure Cr. Based on this observation, many researchers considered that there might be some electrochemical reactions related to the corrosion process. One of the possible explanations is that H_2_O continuously evaporates and subsequently absorbs on the layer of salt [1], which promotes the electrochemical reactions and eventually accelerates the metal s corrosion [2]. However, the mechanism has not yet been confirmed by experiment. To understand the mechanism, firstly, electrochemical measurements including cell testing, use of electrodes, and electrochemical impedance spectroscopy (EIS) were set up by the authors and used to study the electrochemical behavior, which confirmed that the electrochemical reactions occurred during the corrosion of pure Fe under a solid NaCl deposit in a water vapor atmosphere [9]. In addition, the co-reaction between electrochemical and chemical reactions was observed, which confirmed that the corrosion of the metals was accelerated under the salt deposit in the atmosphere of H_2_O + O_2_. However, the corrosion mechanism (*i.e.*, the corrosion reaction) has not yet been studied in detail and further effort is required.

The deposit of sulfates (especially Na_2_SO_4_) on metallic parts which operate under damp air at intermediate or high temperatures is a normal phenomenon, such as occurs with gas turbines and power plants located in marine environments [8]. According to previous studies [1,2,3,4,5,6,7,8], a co-effect between deposited sulfate and H_2_O + O_2_ might exist and significantly accelerate the corrosion of metals in a similar way to that occurring with NaCl + H_2_O + O_2_. However, studies on the synergistic effects of Na_2_SO_4_ + H_2_O + O_2_ on the corrosion of metals or alloys are still lacking. Based on the operating temperature, the sulfate deposit has two different forms, molten and solid. Recently, many studies have been carried out on the corrosion behavior of metals/alloys in a molten Na_2_SO_4_ environment [10,11,12,13,14,15,16] and many corrosion mechanisms have been proposed. One of the well-known mechanisms is the sulfidation model [10,11], in which the formation of sulfides accelerates the corrosion. The other one is the acidic-basic fluxing [10,12,13,14,15] mechanism, in which dissolution of the scale of protective oxides due to formation of basic Na_2_O was considered as the reason for the accelerated corrosion. Moreover, based on the electrochemical mechanism [16], corrosion was considered as an electrochemical reaction in which the transfer of electrons accelerated the corrosion. However, the corrosion mechanism under a solid sulfate deposit at an intermediate temperature (~500 °C) has not yet been widely investigated, without which the development of protection methods for metals or alloys has been restricted.

In order to reveal the corrosion mechanisms of the turbine blades in aeroplanes and ships straightforwardly, the pure metal was used for simplification. In previous studies, the corrosion behavior of pure Fe and Cr under solid NaCl in wet oxygen was studied, which showed a faster corrosion rate compared to only NaCl or wet oxygen alone. However, the effect of solid Na_2_SO_4_ in wet oxygen on stainless steel has not been studied before. Therefore, this work focuses on the corrosion behavior of metals under solid Na_2_SO_4_ in wet oxygen. Normally, the material used for turbine blades in aeroplanes is 1Cr11NiW2MoV steel, in which Fe is a main component. To study the corrosion mechanisms of the pure metal, it is helpful to understand the corrosion behavior of the turbine blades. The main metals in stainless steel are Fe and Cr. Both of these metals need be studied in detail. In this paper, the corrosion behavior of pure Fe under solid Na_2_SO_4_ deposit in an atmosphere of water vapor plus oxygen was investigated firstly by mass gain measurement, EIS, X-ray diffraction (XRD), scanning electron microscope (SEM). The corrosion mechanism is discussed in detail.

## 2. Results

### 2.1. Mass Gain Measurements

**Figure 1:**
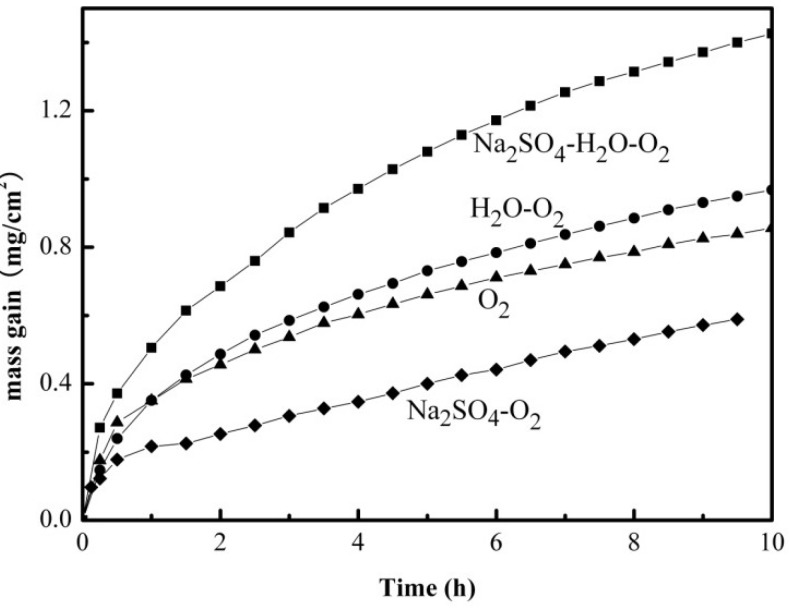
Corrosion kinetics curves of the pure Fe under various corrosion environments at 500 °C: (I) dry O_2_ (▲); (II) H_2_O + O_2_ (●); (III) Na_2_SO_4_ + O_2_ (♦),and (IV) Na_2_SO_4_ + H_2_O + O_2_ (■).

Figure 1 shows the mass gain of the pure Fe as a function of time at 500 °C in four different corrosion environments: (I) dry O_2_; (II) H_2_O + O_2_; (III) Na_2_SO_4_ + O_2_; and (IV) Na_2_SO_4_ + H_2_O + O_2_. As can be seen from Figure 1, the mass gain of the pure Fe under the different environments shows an increasing order of IV < I < II < III, which implies that a Na_2_SO_4_ deposit could inhibit the oxidation process of the pure Fe under certain conditions, particularly in the absence of H_2_O. The highest mass gain was observed when H_2_O + O_2_ was introduced together with Na_2_SO_4_, which suggests that a synergistic effect exists between Na_2_SO_4_ and H_2_O + O_2_ on the corrosion process of the pure Fe, which accelerates the corrosion of the pure Fe at 500 °C.

### 2.2. Electrochemical Corrosion

**Figure 2:**
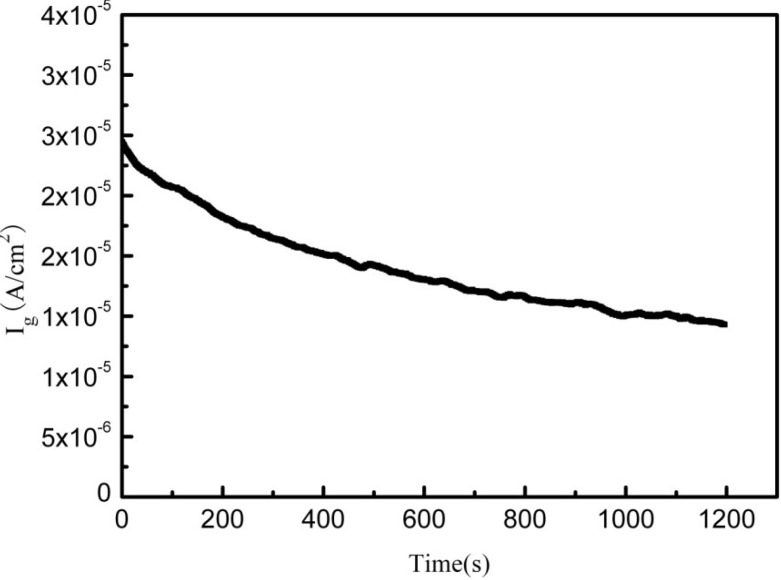
Galvanic corrosion current of the Fe-Pt couple under a Na_2_SO_4_ deposit in an atmosphere of H_2_O + O_2_ at 500 °C.

**Figure 3:**
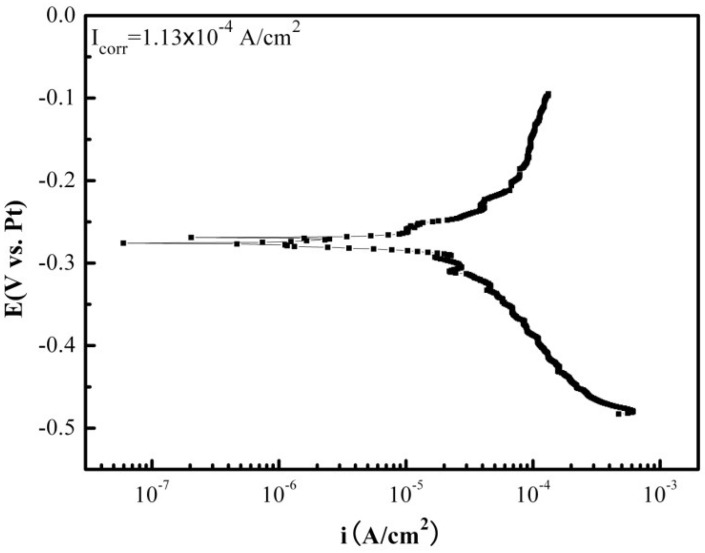
The potentiodynamic polarization plot of the pure Fe under a Na_2_SO_4_ deposit in an atmosphere of H_2_O + O_2_ at 500 °C.

**Figure 4:**
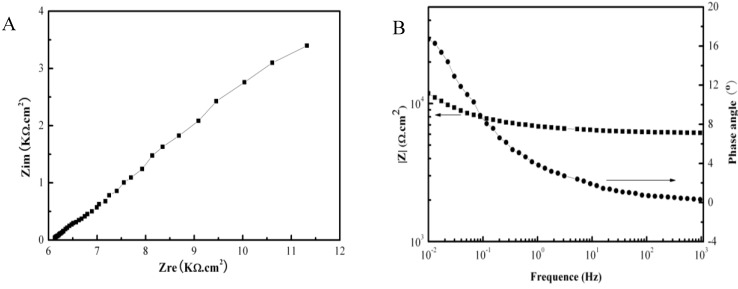
EIS measurement results of the pure Fe under a Na_2_SO_4_ deposit in an atmosphere of H_2_O + O_2_ after 1 h oxidation at 500 °C: (**A**) Nyquist plot; (**B**) Bode plot.

**Figure 5:**
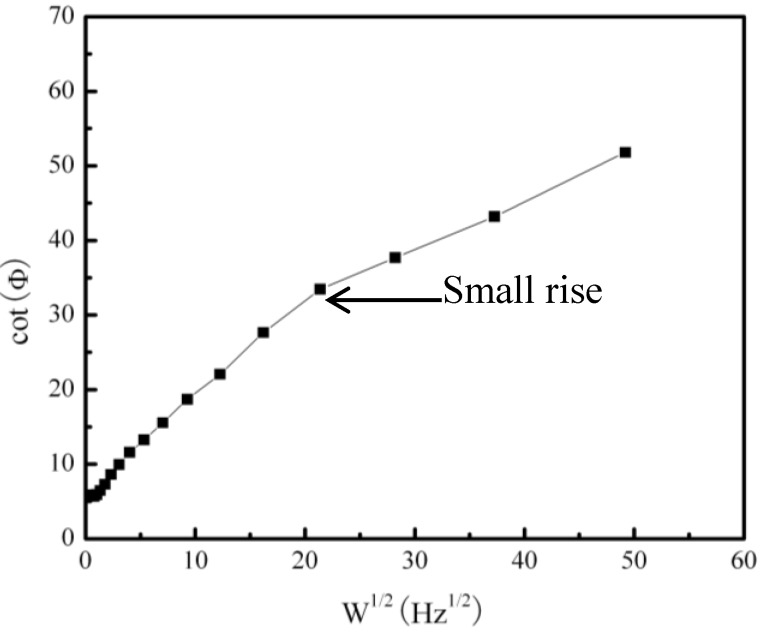
Frequency dependence of the phase angle of the pure Fe under a Na_2_SO_4_ deposit in an atmosphere of H_2_O + O_2_ at 500 °C at open circuit potential.

**Figure 6:**
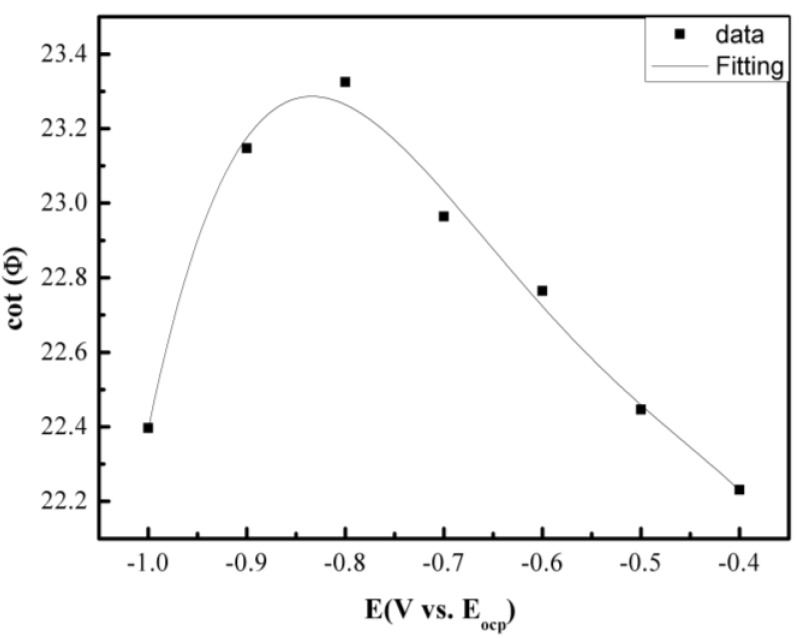
Cathodic potential dependence of the phase angle of the pure Fe under a Na_2_SO_4_ deposit in an atmosphere of H_2_O + O_2_ at 500 °C.

Figure 2 shows the galvanic corrosion current (*I*_g_) as a function of time for a couple of Fe-Pt under a solid Na_2_SO_4_ deposit with an atmosphere of H_2_O + O_2_ at 500 °C. The result shows that the *I*_g_ decreases from about 3 × 10^−5^ A/cm^2^ to 1 × 10^−5^ A/cm^2^ with an increase in time from 0 to 1200 s. Figure 3 shows the potentiodynamic polarization curves of the pure Fe under the testing environment. As can be seen, the anodic current density linearly increases with increases in the anodic potential in the active polarization zone, which can be attributed to the active dissolution of the metal. Figure 4 shows an *in situ* EIS result of the pure Fe after 1 h corrosion under the testing environment. It shows two time constants in the Nyquist plot. The capacitances correspond to the electrochemical reaction and the formation of oxidation products on the surface of the electrode at high and low frequencies, respectively [8]. Figure 5 shows the frequency dependence of the phase angle of the pure Fe with the Na_2_SO_4_ deposit and the H_2_O + O_2_ at 500 °C at open circuit potential. Figure 6 shows the cathodic potential dependence of the phase angle of the pure Fe under the Na_2_SO_4_ deposit and the H_2_O + O_2_ at 500 °C. As can be seen, the potential E corresponding to the maximum of cotΦ is negative to E_1/2_ (semiwave potential).

### 2.3. Corrosion Products and Morphologies

**Figure 7:**
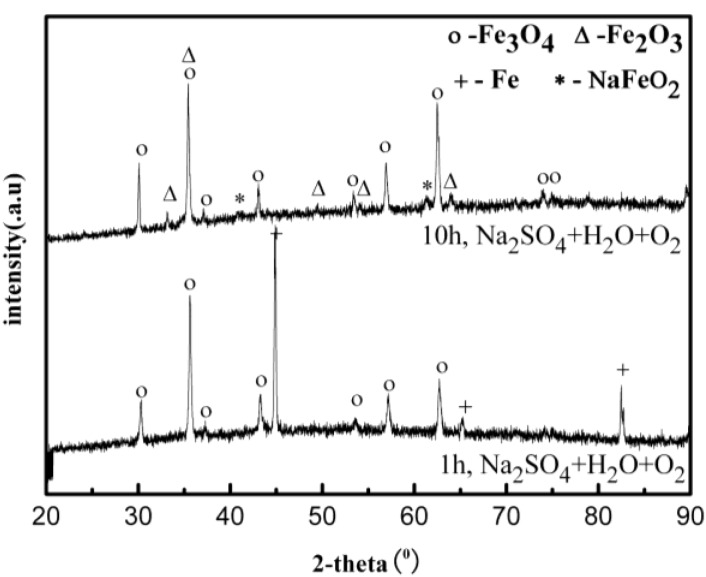
X-ray diffraction (XRD) results of the corrosion scale of the pure Fe under a Na_2_SO_4_ deposit in an atmosphere of H_2_O + O_2_ at 500 °C.

**Figure 8:**
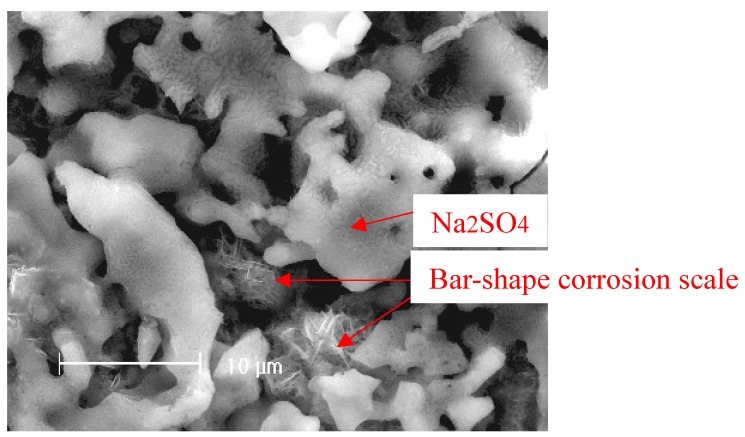
The morphology of the Na_2_SO_4_ film on the surface of the pure Fe after corrosion under a Na_2_SO_4_ deposit in an atmosphere of H_2_O + O_2_ for 10 h.

After corrosion tests, the samples were analyzed by XRD after cleaning with distilled water. Figure 7 shows the XRD patterns of the corrosion scale formed on the surface of the Fe in the Na_2_SO_4_ + H_2_O + O_2_ environment after 10 h. The results show that the corrosion scale is composed of Fe_3_O_4_, Fe_2_O_3_, and NaFeO_2_. Figure 8 shows an example of SEM surface morphology of the corroded sample with a deposited Na_2_SO_4_ layer in a H_2_O + O_2_ atmosphere at 500 °C after 10 h. It can be seen that the Na_2_SO_4_ layer turned from an originally thick and compact layer to a loose and porous one after 10 h. The corrosion scale with a bar-shape appears underneath the Na_2_SO_4_ layer. Obviously, Na_2_SO_4_ participates in the corrosion reaction of the pure Fe.

**Figure 9:**
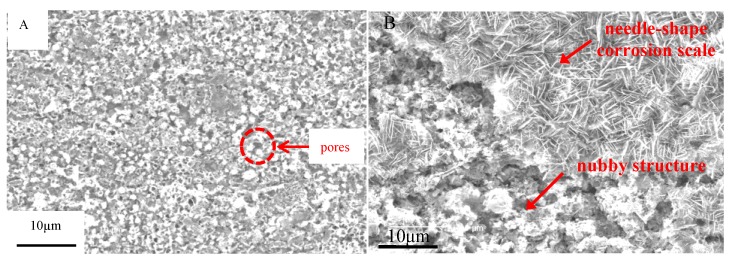
Surface morphologies of the pure Fe under a Na_2_SO_4_ deposit in an atmosphere of H_2_O + O_2_ after different corrosion times: (**A**) 1 h; (**B**) 10 h.

**Figure 10:**
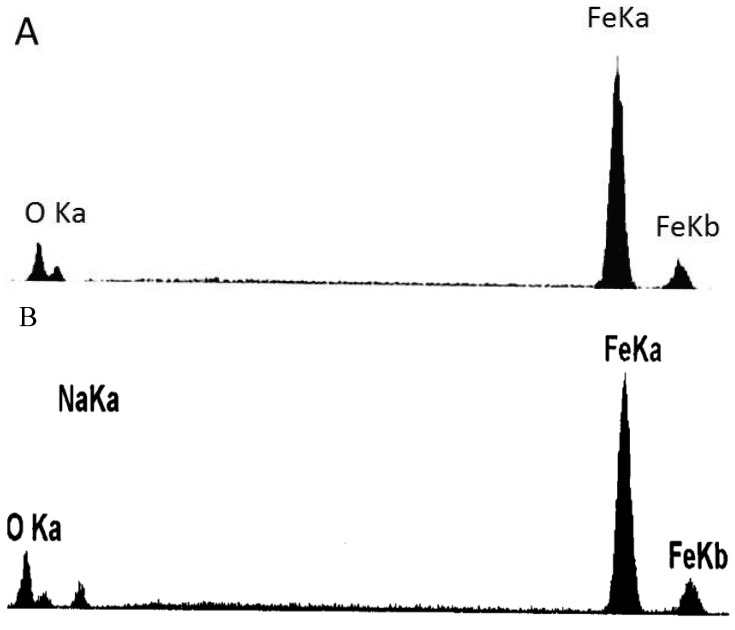
Energy dispersive X-ray spectroscopy (EDAX) results of the products formed on the pure Fe under a Na_2_SO_4_ deposit in an atmosphere of H_2_O + O_2_ after corrosion for 10 h: (**A**) nubbly-shape products; and (**B**) needle-shape products.

Figure 9 shows the SEM morphologies of the corrosion scale on the surface of the pure Fe after corrosion for 1 h and 10 h in the Na_2_SO_4_ + H_2_O + O_2_ environment at 500 °C after the Na_2_SO_4_ residual layer has been removed. The corrosion scale is uniform after corrosion for 1 h. The corrosion product is porous, which may be produced by the formation of volatile products [2]. However, the product scale changes to be uneven after corrosion for 10 h. In addition, the morphologies of needle- and nubbly-shape appear which are not seen in the case after 1 h. From the corresponding EDAX results (see Figure 10), the needle-shape product is composed of Fe, Na, and O elements, while Fe and O are the only two elements detected in the nubbly structure. According to the XRD and the previous research results [17], the needle-shape product is most likely a mixture of Fe_2_O_3_ and NaFeO_2_ and the nubbly product may be Fe_3_O_4_. Figure 10 shows the cross-sectional morphologies of the corrosion scale on the pure Fe after corrosion for 1 h and 10 h under a Na_2_SO_4_ deposit in a H_2_O + O_2_ atmosphere at 500 °C. After 1 h, the layer of corrosion products is thin, porous, and cracked. After 10 h, the product scale is entirely different from that at 1 h, *i.e.*, the outer part of the product scale has a lot of pores; the inner part of the scale is comparatively compact but has some micro-cracks.

## 3. Discussion

### 3.1. The Interaction between Electrochemical Corrosion Reaction and Chemical Corrosion Reaction

The galvanic corrosion current measurement reveals that the I_g_ of the coupled Fe-Pt under a Na_2_SO_4_ deposit in an atmosphere of H_2_O + O_2_ at 500 °C is about 10^−5^ A/cm^2^ (Figure 3). It is believed that no electrochemical reaction occurs on Pt-Pt electrodes under the tested corrosion environment and its corresponding tested current density is near to zero [9]. Compared to the Pt-Pt electrodes, the appearance of current density in the Fe-Pt electrodes supplies the required information as it is produced by electrochemical reactions. Thus, electrochemical reactions are considered to be involved in the corrosion of the pure Fe under the tested environment. The entire corrosion reactions including the electrochemical corrosion reaction and the chemical oxidation reaction are the same as the results obtained under the solid NaCl deposit in an atmosphere of H_2_O + O_2_ at 600 °C [18]. The percentage of the electrochemical corrosion to the total corrosion was calculated, because it provides a useful insight into the corrosion behavior of the pure Fe under a solid Na_2_SO_4_ deposit in an atmosphere of H_2_O + O_2_ at 500 °C. The total corrosion rate can be calculated by mass gain measurements from the consumption of Fe over the entire corrosion. Since the oxidation products are mainly composed of the oxygen involved in the corrosion process and the metal consumed by corrosion, which is approximately Fe_2_O_3_, then the ratio of atoms is assured. The total amount of the consumed metal (Fe) can be determined from the mass gain results, which is calculated from the total Fe_2_O_3_ amount according to the ration of atoms. The electrochemical corrosion rates (*I*_corr_) can be calculated by fitting the potentiodynamic polarization curves in the anodic active polarization zones. The calculation method is described in detail in the following reference [18]. In this study, the calculated percentage of the electrochemical corrosion to the total corrosion is about 1.4%, which is low and means the electrochemical corrosion is weak during the entire corrosion process.

In previous studies [9,18], the percentages of the electrochemical corrosion to the total corrosion of the pure Fe and the pure Cr are also small under a solid NaCl deposit in an atmosphere of H_2_O + O_2_ at intermediate temperature. However, the following study reveals that the electrochemical corrosion reaction couples to the preceding chemical corrosion reaction, which significantly accelerates the corrosion. According to the results, it is clear that the corrosion rate of the Fe under the environments of Na_2_SO_4_ alone, or H_2_O alone, is significantly lower than that under both Na_2_SO_4_ + H_2_O. The results show that with salt alone or H_2_O alone, only the chemical reaction occurs [18]. While under the synergistic effect of salt and H_2_O, the electrochemical and chemical reaction will co-effect on the corrosion [18]. Therefore, it can be concluded that the occurrence of the electrochemical corrosion reaction promotes the progress of the chemical corrosion reaction, which sharply accelerates the corrosion rates of the pure Fe and the pure Cr [18]. Also, from Figure 2, the corrosion rate of the pure Fe is dramatically accelerated under the solid Na_2_SO_4_ deposit in the water vapor, which implies that the corrosion rate of the pure Fe may be accelerated due to the introduction of an electrochemical corrosion reaction.

From the EIS results, the corresponding electrochemical parameters may be obtained. Among them, Φ is the phase angle and ω is the angle frequency [18]. It is well-know that different mathematic calculations for these parameters have different physical meanings. According to Smith [19], a plot of cotΦ *vs.* ω^1/2^ can be used for mechanistic diagnosis. For example, an electrochemical reaction coupled to either a preceding chemical reaction (ce) or a following chemical reaction (ec) will show a maximum in a plot of cotΦ *vs.* ω^1/2^, while a catalytic reaction (ec´) will give cotΦ close to infinity as ω is close to zero, where c´ is a catalytic reaction. If the electrochemical reaction does not couple to a chemical reaction, the plot of cotΦ *vs.* ω^1/2^ will show a straight line. The frequency dependence of the phase angle of the pure Fe under a Na_2_SO_4_ deposit in an atmosphere of H_2_O + O_2_ at 500 °C at the open circuit potential is shown in Figure 5. The result shows that it is not a straight line and has a maximum. Therefore, the coupled effect of the electrochemical reaction and the chemical reaction can be confirmed.

However, the plot of cotΦ *vs.* ω^1/2^ cannot be used to distinguish between these two mechanisms but the plot of cotΦ *vs.* (*E* − *E*_1/2_) is required. For a chemical reaction in which the reaction rate constants *k*_1_ = *k*_2_, from the ce mechanism, the potential *E* corresponding to the maximum of cotΦ is negative to *E*_1/2_; whereas for ec, the potential *E* corresponding to the maximum of cotΦ is positive to *E*_1/2_. For a chemical reaction in which *k*_1_ ≠ *k*_2_, from the ce mechanism, the cotΦ decreases with increasing (*E* − *E*_1/2_); whereas for an ec mechanism, the cotΦ increases with increasing (*E* − *E*_1/2_) [19]. Figure 6 shows the cathodic potential dependence of the phase angle of the pure Fe under a Na_2_SO_4_ deposit in the atmosphere of H_2_O + O_2_ at 500 °C. The result indicates that the cotΦ increases with increasing (*E* − *E*_1/2_), which means that corrosion mechanisms are consistent to the ce mechanism.

### 3.2. The Corrosion Behavior of the Pure Fe under a Na_2_SO_4_ Deposit in an Atmosphere of H_2_O + O_2_

**Figure 11:**
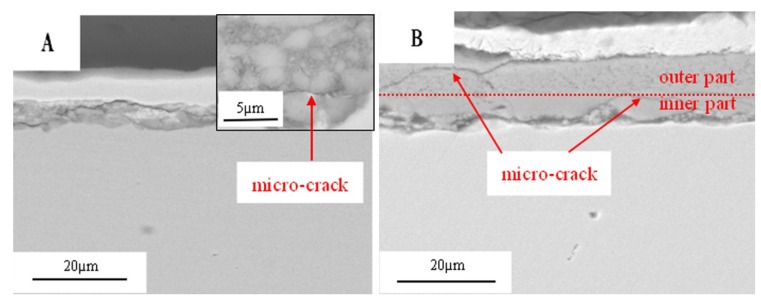
Cross-sectional morphologies of the corrosion scale of the pure Fe under a Na_2_SO_4_ deposit in the atmosphere of H_2_O + O_2_ after corrosion for 1 h and 10 h.

The significantly increased corrosion rate of the pure Fe under a Na_2_SO_4_ deposit in the presence of H_2_O suggests that the effect of H_2_O on the corrosion is important and cannot be neglected. The formation of the corrosion scale with the porous layer (Figure 9A) may be due to the formation of volatile products during the corrosion process [8]. Figure 11 shows the cross-sectional morphologies of the corrosion products. The introduction of the H_2_O may promote the formation of H_2_ [20], which is one of the hypotheses that is not yet experimentally confirmed. However, the porous product scale confirms the formation of the volatile products. Based on this and previous study results [1,2,3,4,5,6,7,8,9,18], the following corrosion mechanism is proposed: at initial stages of the corrosion, the Na_2_SO_4_ and the H_2_O can react with the Fe to produce NaFeO_2_ and H_2_SO_4(g)_:

4Fe + 3O_2_ + 2H_2_O +2Na_2_SO_4_ → 4NaFeO_2_ + 2H_2_SO_4_(g) (1)

Based on the above results and discussion as well as our previous study [9,17], in the electrochemical corrosion reaction coupled with a chemical corrosion reaction, the H_2_SO_4_ would electrochemically react with the Fe.

Cathodic reaction: H_2_SO_4_ + 2e^-^ → SO_4_^2-^ + H_2_ (2)

Anodic reaction: Fe − 2e^-^ → Fe^2+^ (3)

From these reactions, H_2_SO_4_ can be consumed by the cathodic reaction. Subsequently, the chemical reaction of (1) will be promoted in the forward direction. The electrochemical reaction can promote the progress of the chemical reaction, which will accelerate the corrosion rate of the pure Fe.

FeSO_4_ can also react with the H_2_O and the O_2_ to form iron oxides and H_2_SO_4_(g) [6,8]:

6FeSO_4_ + O_2_ + 6H_2_O → 2Fe_3_O_4_ + 6H_2_SO_4_(g) (4)

5FeSO_4_ + O_2_ + 5H_2_O → Fe_3_O_4_ + Fe_2_O_3_ + 5H_2_SO_4_(g) (5)

The produced H_2_SO_4_(g) will cyclically react with the Fe, and continuously accelerate the corrosion process.

Meanwhile, during the corrosion process, the pure Fe would be oxidized by the following reactions by the H_2_O and the O_2_ in addition to the above mechanism [1]:

2Fe + 2H_2_O + O_2_ → 2Fe(OH)_2_ (6)

6Fe(OH)_2_ + O_2_ → 2Fe_3_O_4_ + 6H_2_O (7)

### 3.3. Thermodynamic Calculation

The Gibbs criterion is a useful tool to estimate whether a chemical reaction can take place or not. To start a reaction, the change of Gibbs free energy must satisfy Equation (8) below:

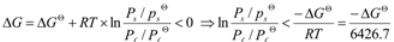
(8)

*P_s_*, *P_s_*^Θ^, *P_f_*, *P_f_*^Θ^, and Δ*G*^Θ^ stand for partial pressure of products, standard partial pressure of products, partial pressure of reactants, standard partial pressure of reactants, and standard Gibbs free energy, respectively [1,2,3,4,5,6,7,8].

To calculate Gibbs free energy, two hypotheses are given as: (1) the air involved in the corrosion is exclusively the H_2_O and the O_2_; (2) the partial pressure of the H_2_O (47,307.1 Pa) and the O_2_ (53,992.9 Pa) at the interface of the pure Fe and the Na_2_SO_4_ are similar to that in the furnace. The calculated results, listed in Table 1, show that the corrosion reactions are favorable on thermodynamic considerations under the experimental conditions. The quantity of H_2_SO_4_ in the tested conditions is much lower than the theoretically thermodynamic value, and thus the deduced corrosion mechanism is supported by the thermodynamic calculations.

**Table 1:** Thermodynamic calculation results.

Reactions	Conditions
4Fe + 3O_2_ + 2H_2_O + 2Na_2_SO_4_ → NaFeO_2_ + 2H_2_SO_4_	P_H2SO4_ < 19,090.3 Pa
H_2_SO_4_ + Fe → FeSO_4_ + H_2_	P_H2_ < 1.02 P_H2SO4_
6FeSO_4_ + O_2_ + 6H_2_O → 2Fe_3_O_4_ + 6H_2_SO_4_	P_H2SO4_ < 42,116.9 Pa
5FeSO_4_ + O_2_ + 5H_2_O → Fe_2_O_3_ + Fe_3_O_4_ + 5H_2_SO_4_	P_H2SO4_ < 41,040.8 Pa
4Fe + 3O_2_ + 2Na_2_SO_4_ → 4NaFeO_2_ + 2SO_3_	P_SO3_ < 42,491.1 Pa

## 4. Experimental Section

### 4.1. Materials and Experimental Conditions

In this work, pure Fe (99.9%) was employed as the experimental specimen. Before experiments, the sample was cut into coupons with a dimension of 10 mm × 15 mm × 2 mm, ground with silicon-carbide abrasive paper finally to 1000 grit, degreased in acetone and ethanol, and then dried in air. Na_2_SO_4_ was deposited on the preheated Fe coupon surface by repeatedly brushing and drying with a Na_2_SO_4_-saturated solution. The mass of the Na_2_SO_4_ was about 4 mg/cm^2^.

All corrosion experiments were carried out at 500 ± 10 °C. The temperature of the water bath to produce H_2_O vapor was fixed at 80 °C (46.8 vol%) and the flux of the O_2_ was 200 mL/min.

### 4.2. Mass Gain Measurements

The corrosion tests were carried out in a thermal balance [1,2,3,4,5,6,7,8]. Water (H_2_O) came from an 80 °C water bath. Pure O_2_ was passed through the glass bubbler with a flux of 200 mL/min. To prevent condensation of the water vapor in the upper part of the thermal balance, a counter-flow of N_2_ was passed through the apparatus at 150 mL/min. After the furnace was heated to the desired temperature and the gas flow was stabilized, the specimen was quickly suspended into the furnace tube, and the test was started. All the measurements were carried out at ambient pressure. There were five parallel samples for each test.

After the tests, the specimens were further examined by XRD and SEM/EDX.

### 4.3. Electrochemical Experiments

**Figure 12:**
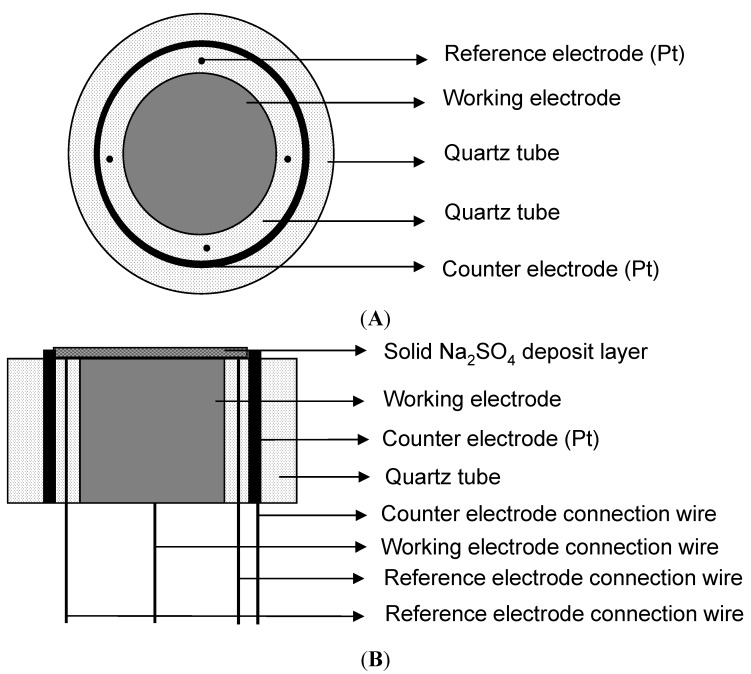
Schematic diagram of three-electrode cell: (**A**) the top view; (**B**) the cross-section view.

A special three-electrode system was built for the electrochemical measurements in this particular environment (shown in Figure 12) [9]. To decrease the resistance of the solution and get a uniform electric field, the reference electrodes consisted of four platinum wires with a diameter of 0.4 mm, and the counter electrode was a circular strip of platinum foil with a width of about 2 mm. All potential values in this paper were reported *versus* the platinum reference electrode. The Fe working electrode was a rod with a length of 10 mm and a diameter of 5 mm. The three electrodes were in quartz tubes, which acted as insulators. All the gaps were sealed by high temperature inorganic glue. The three-electrode system after the solid Na_2_SO_4_ deposition was directly put into the furnace at the desired temperature with the water vapor for electrochemical measurements.

The PAR2273 Electrochemical Measurement System manufactured by EG&G was used for all electrochemical measurements, which also has the function to compensate the resistance between reference electrode and working electrode. In the galvanic corrosion measurement, the ratio of anodic area to cathodic area is 1:2. In EIS measurements, the AC perturbation was 0.1 V because of the low conductivity of Na_2_SO_4_ at 500 °C [21,22] and the frequency was swept from 10 kHz to 10 mHz. The resistance between reference and working electrodes was compensated during measurements according to the design of the electrochemical system and testing work station. The measurements were repeated more than three times.

## 5. Conclusions

The corrosion rate of the pure Fe is significantly accelerated under a Na_2_SO_4_ deposit in an atmosphere of H_2_O + O_2_ at 500 °C. The introduction of H_2_O improves the formation of the NaFeO_2_ and the H_2_SO_4_, which accelerates the chemical reactions during the entire corrosion. The occurrence of the electrochemical corrosion reaction promotes the progress of the chemical corrosion reaction. Both of them accelerate the corrosion of the pure Fe. Also, the deduced corrosion reactions were confirmed by thermodynamic calculation.
